# Vital Legacy

**DOI:** 10.17533/udea.iee.v43n3e16

**Published:** 2025-11-06

**Authors:** Juan Guillermo Rojas

**Affiliations:** 1 Nurse, PhD. Professor. Dean of the Faculty of Nursing at Universidad de Antioquia, Colombia. Email: guillermo.rojas@udea.edu.co. Corresponding author. https://orcid.org/0000-0002-0255-4344 Universidad de Antioquia Faculty of Nursing Universidad de Antioquia Colombia guillermo.rojas@udea.edu.co

In 1950s Medellín, the city had nearly 358,000 inhabitants due to migratory flows from the towns of Antioquia into the capital, gradually converting the noble city into a center of industrial development.^1^

By then, the growing social needs motivated the design of the first pilot plan for the city’s urban improvement by the Public Improvement Society, which comprised controlled expansion of urban settlements towards the slopes, channeling of the Medellín River, construction of an administrative center, and consolidation of an industrial zone. Gradually, the colonial architecture was replaced by modern construction with Art-Deco designs, which gave an *avant-garde* touch to the growing and prosperous city of eternal spring.^2^ The expansion and urban and population growth brought along other challenges for the city: one was to improve basic sanitation infrastructure, and the other, to effectively respond to the needs of public health care and hospital care with nurses trained in basic sciences, medical sciences and pathology, medications, socio-humanistic aspects, and vast ethical and moral sufficiency. 

This was the context in which Doctor Ignacio Vélez Escobar, as Dean of the Faculty of Medicine at Universidad de Antioquia, proposed the creation of the School of Nursing with support from the Dominican Sisters of Charity of the Presentation of Tours, beginning a new chapter in the history of nursing care in Antioquia and Colombia, which in the words of our Professor Cecilia Mabel Restrepo, set the guidelines and laid the foundations for the evolution and development of the profession in our environment.^3^

The historical development of the Faculty of Nursing, as an academic project, has been permeated by changes and social, economic, and political transformations, as well as by the historical events within the profession, which - as indicated by María Victoria López - evidence the learnings from the processes of epistemological, theoretical and practical reflection that have strengthened the profession,^4^ and which have served to search for new ways to understand human experiences and propose strategies for formation. This, in the words of Siles, implies that both nursing and care constitute a historical-anthropological reality that categorically reflects the cultural and historical nature of the discipline^5^ and the profession in response to the needs for individual care, collective care, and common home care and which mark new pathways for professional training. 

It is precisely the capacity to adapt and transform the training over time, which has characterized the Faculty of Nursing, evidencing that social development is manifested in professional curricula, which respond to the demands of the changes that have arisen and the social, cultural, political and economic requirements in which the disciplines develop.^6^

Celebrating the 75 years of the Faculty of Nursing represents the vital legacy that today calls us to re-elaborate and dimension the past history, to understand the present reality of the profession and propose the utopias of the future.^3^

This vital legacy that connects us with history, which, like culture, is configured as a web of symbols and meanings that according to Geertz,^7^ must be known and interpreted as the context to understand the social phenomena that describe the profession’s cultural history and the symbols that identify it. 

The history of the Faculty of Nursing has been constructed from the framework of the history of thousands of men and women who have assumed their commitment to human care, transcending practical facts and actions to a philosophy of life that crosses borders and surpasses the limits of time, leaving a mark and setting the course for the future. This is what Professor Cecilia Mabel Restrepo has referred to, calling on us to recover the collective memory of nursing, which allows revealing the meanings embedded in the fabric of the collective unconscious, which permits finding and understanding that which prevails in the minds of contemporary actors.

In 75 years, many moments, events, and stories have taken place, which is why it is necessary to recognize our teachers of care, both religious and lay; those who accompany us today and those who have already transcended the earthly plane, they all bravely took on the challenge of laying the foundations on which the current academic project is based. The humanistic, *avant-garde*, and resilient thinking of these teachers has made it possible to overcome the critical moments that both the University and the Faculty have experienced.

Today, it is essential to specially recognize the professors and students, as protagonists of the present of the Faculty, which faces multiple challenges in the current context of political and financial crisis the country is going through. However, their impetus and critical analysis are the promise of value to find the best alternative solutions to maintain a public, open, and functioning university. 

Similarly, the training work has been possible due to the effort and dedication of our administrative support and maintenance collaborators; their life stories and struggles have also taught us to live care as a transformative experience built through teamwork.

We recognize our graduates who are the living image of the University in society and connect the philosophical, theoretical, and scientific postulates of the discipline with the reality of practice under the university values ​​and principles. Today, our graduates contribute to care in different scenarios and stand out for their high level of social sensitivity. They are even part of the diaspora of nursing knowledge that has spread throughout the world, contributing to care in other countries with high levels of humanism and quality. 

The history of our Faculty is also a history of transformation, like a river that flows forth and widens over the years, the conversion from School to Faculty, the expansion of research and extension, the emergence of the Journal *Investigación y Educación en Enfermería*, the creation of the Research Center, the creation and recognition of five research groups, the creation of master’s in interdisciplinary Collective Health, the development of four clinical specializations, a disciplinary master’s and a PhD, which have given thousands of nursing professionals and related professions the opportunity to expand their skills into specialized fields that have positively impacted care in clinical, community, and decision-making settings. 

Within this transformation process, the Faculty of Nursing has ventured into managing health intervention projects, public policy management, support for caregiver networks, volunteer actions in health and lifelong education through the mission axis of extension, sealing the commitment to university social responsibility. 

In the construction of this historical path, the character of nursing as a professional discipline of a practical nature imposes a series of challenges for training in the future: to consolidate an academically and socially relevant quality training program, that is, to respond to local and global health challenges with a high sense of humanism, with an interprofessional approach, which aims to care for the planet, that responds efficiently to the expansion of advanced nursing practice and that helps in the consolidation of a just and peaceful society. 

Likewise, consolidating the new forms of formative interaction through the use of ICTs by strengthening the virtual campus and the use of innovative strategies, bringing the responsible use of emerging technologies and artificial intelligence closer to the care service. Also, advancing on an inclusive formation that allows youth from the region and the country to access quality public university education as a way of democratizing knowledge and promoting social development. 

Another challenge is to continue expanding research as a means to preserve and expand disciplinary knowledge and as a basis to improve training processes in response to the needs of the context. This Faculty has been and expects to remain the scenario for individual and collective reflection and creative thinking that continues enhancing nursing. 

In synthesis, the historical development of the Faculty of Nursing must not be seen as a sum of facts or events, but rather as an integrative vision of history that contributes to strengthening the collective awareness and identity around the disciplinary object as an essential service for humanity.^5^

Facing the 21^st^ century, we assume the challenge of continuing the vital legacy that connects the past, present, and future with the conviction that Universidad de Antioquia and, particularly, the Faculty of Nursing will continue to be a “beacon of light” for society in the search to improve care for all society. 

We honor the past, care for the present, shape the future
